# Alexithymia and interpersonal problems in healthy young individuals

**DOI:** 10.1186/s12888-023-05191-z

**Published:** 2023-09-21

**Authors:** Pauline Koppelberg, Anette Kersting, Thomas Suslow

**Affiliations:** Department of Psychosomatic Medicine and Psychotherapy, University of Leipzig Medical Center, Semmelweisstr, 10, 04103 Leipzig, Germany

**Keywords:** Alexithymia, Interpersonal problems, Difficulties describing feelings, Difficulties identifying feelings, Agency, Communion

## Abstract

**Background:**

Alexithymia refers to a multidimensional personality trait with the facets difficulties identifying feelings (DIF), difficulties describing feelings (DDF), and externally orientated thinking (EOT). Alexithymia is a risk factor for mental and somatic disorders. Previous research with patients suffering from various disorders showed positive relationships between alexithymia and interpersonal problems. Only one study analyzed the link between alexithymic features and interpersonal difficulties in healthy individuals but yielded inconclusive findings because participants’ negative affects were not controlled. A widely accepted conceptualization of interpersonal problems relies on the interpersonal circumplex, which is defined by two orthogonal dimensions, agency and communion. In the present study, we analyzed which facets of alexithymia are associated with the two interpersonal problem dimensions and the global severity of interpersonal distress, after adjusting for negative affect.

**Methods:**

Two-hundred healthy young individuals (100 women) participated in the study. Alexithymic features were assessed using the 20-Item Toronto Alexithymia Scale (TAS-20). Interpersonal problems were measured with the Inventory of Interpersonal Problems (IIP-D). Participants’ state and trait anxiety, depressive symptoms, and verbal intelligence were also assessed.

**Results:**

All alexithymia scales were positively correlated with general interpersonal distress. Regression results suggested that the TAS-20 subscale DIF was the primary predictor of general interpersonal distress after controlling for negative affectivity. The scale DDF correlated negatively with the IIP-D dimension agency. According to our regression analysis, DDF was a predictor of (low) agency controlling for negative affects. Moreover, DDF correlated negatively with the IIP-D dimension communion. Our regression results indicate that DDF was a predictor of (low) communion independent of negative affect. Correlations between alexithymia facets and IIP-D subscales did not differ between genders.

**Conclusions:**

Difficulties identifying feelings seem to be linked to a high level of general interpersonal distress. Difficulties in recognizing one’s feelings may disrupt emotion regulation, which could heighten the general risk of interpersonal problems. Difficulties describing feelings could be a central factor contributing to interpersonal problems related to low communion as well as low agency, since emotion expression and communication are crucial in establishing experiences of social closeness and directing other people’s behavior.

**Supplementary Information:**

The online version contains supplementary material available at 10.1186/s12888-023-05191-z.

## Background

The term *alexithymia* stems from the Greek, literally meaning ‘lacking words for feelings’ [1] and refers to a multidimensional personality trait with the main facets difficulty identifying feelings and differentiating them from the bodily sensations, difficulty describing feelings to others, and an externally orientated cognitive style [2–4]. Currently, the most widely used measure to assess these alexithymic characteristics is the 20-Item Toronto Alexithymia Scale (TAS-20 [5, 6]). Alexithymia is considered to be an important vulnerability factor for the development of psychological distress, mental disorders, and physical illness [7, 8]. It is considered a transdiagnostic, non-specific feature in many mental disorders [9]. There is evidence that alexithymic characteristics occur more frequently in men, elderly people, and individuals with low socioeconomic status or low educational level [10, 11]. About 10% of the general population manifests clinically relevant alexithymia [12, 13]. For young women, the prevalence rate of clinically relevant alexithymia could be lower (about 5%) [14]. Relative and absolute stabilities were found to be high for alexithymia in non-clinical populations, even for long follow-up periods [15]. There is considerable debate about the etiology of alexithymic features. Besides biological models, which associate alexithymia, for example, with dysfunctions of specific brain structures [16, 17] or genetic factors [18, 19], it has been argued that alexithymia may develop as a reaction to early life stress and traumatic events [20, 21].

Already in the early years of clinical alexithymia research it was observed that alexithymia is concomitant with interpersonal problems. Nemiah and Sifneos [22], for example, noticed that alexithymic patients typically remain detached, indifferent, and distant with others, burdening therapeutic relationships. Marty and M’Uzan [23] reported interpersonal deficits such as chaotic interpersonal relations in psychosomatic patients with difficulties processing emotions. Although alexithymia was defined by intrapersonal deficits, early observations also highlighted interpersonal deficits.

In the last decades, research based on standardized assessment measures has started to systematically investigate the associations between alexithymia and interpersonal problems. In this research, the Inventory of Interpersonal Problems (IIP [24, 25]) was often administered, an internationally used self-report questionnaire assessing interpersonal impairment in clinical contexts. From a general perspective, interpersonal problems are recurrent difficulties, which people exhibit in relating to others [26]. Efforts to conceptualize interpersonal problems have relied primarily on the interpersonal circumplex, which is a model for assessing, and integrating interpersonal traits, motives, and behaviors [27]. The interpersonal circumplex is defined by two orthogonal dimensions or axes, *agency* or *social dominance* and *communion* or *social closeness* [28]. Agency refers to an individual’s control of others and includes traits such as assertiveness, dominance, and independence. It seems relevant to negotiating social hierarchies. Communion relates to involvement with others and includes traits such as cooperation, caring, and friendliness. Communion seems relevant to negotiating interpersonal distance. The IIP follows the circumplex model of interpersonal functioning with two bipolar axes and comprises eight specific domains of interpersonal problems: domineering, vindictive, cold, socially avoidant, non-assertive, exploitable, overly nurturant, and intrusive [24, 25].

To our knowledge, eight studies have been conducted hitherto examining the relationship between alexithymia and interpersonal problems [29–36]. Most of these studies examined samples of patients suffering from various mental [29–33] or physical disorders [35]. Two studies recruited patients and healthy individuals [34, 36] but only Weinryb et al. [36] analyzed the link between alexithymic features and interpersonal difficulties in healthy individuals. Overall, across studies, most but not all reported correlations between alexithymia and interpersonal problems were positive. The global level of alexithymia (i.e., the total alexithymia score) was found to be related most consistently to the interpersonal problem domains cold [29, 32–36] and non-assertive [29, 32–34]. These findings indicate that individuals scoring generally high in alexithymia tend to have problems in connecting with and feeling close to other people and manifest difficulties in taking initiative in relation to others and coping with social challenges.

Even though many studies have examined alexithymia as a unitary construct (i.e., using the total score), recent research underlines the importance of considering the alexithymia facets separately when investigating cognitive-emotional functioning, i.e., behavior and processes of attention, appraisal, memory, and language [37, 38]. Eight studies examined the relationship between alexithymia and interpersonal problems [29–36] of which only five studies conducted analyses at the subscale level [29, 31, 33, 35, 36]. Importantly, results between studies differed considerably: difficulties identifying feelings was positively correlated with interpersonal problem domains in the studies of Spitzer et al. [33], Vanheule et al. [35], Hermes et al. [29] and Ogrodniczuk et al. [31] and with the total score of the IIP in the studies of Spitzer et al. [33], Hermes et al. [29] and Ogrodniczuk et al. [31]. In contrast, difficulties describing feelings correlated positively with interpersonal problem domains (cold and socially avoidant) in the studies of Spitzer et al. [33], Hermes et al. [29] and Weinryb et al. [36] and with the total score of the IIP in the studies of Spitzer et al. [33], and Hermes et al. [29]. A negative correlation between difficulties describing feelings and the scale intrusive was reported by Weinryb et al. [36]. Finally, externally oriented thinking showed positive correlations with the scales cold and vindictive in the studies of Vanheule et al. [35] and Weinryb et al. [36] and negative correlations with the scales exploitable and overly nurturant in the investigation of Vanheule et al. [35]. No correlations were observed between externally oriented thinking and the total score of the IIP. It is interesting to note that the findings of the only study based on a sample of healthy individuals [36] differ substantially from the results reported in the patient-based investigations. The presence of a mental (or physical) disorder could have an effect on severity and type of alexithymia and interpersonal problems and change its relationship. An important methodological limitation of many of the above-mentioned studies is the failure to assess negative affects such as anxiety and depression of study participants. Only three studies measured and controlled participants’ negative affectivity in some way (general psychological distress [33], negative affect [30], or depressive symptoms [31]). On the one hand, negative affects frequently accompany experiences of interpersonal failure and may further intensify relationship problems [39, 40]. On the other hand, anxiety and depressed mood have been found to be linked to heightened levels of alexithymia [17, 41]. Thus, control of participants’ negative affects seems essential to reach clear conclusions on the relationship between alexithymia and interpersonal problems.

In the present study, we explored the relationship between alexithymia facets and interpersonal problems in a sample of healthy individuals. The results of the only previous study based on a sample with healthy individuals are inconclusive since participants’ negative affects were not considered in the analyses. In our study, we assessed participants’ state and trait anxiety and level of depressive symptoms and recruited an equal number of men and women. It should be noted that, besides negative affects, gender is also a variable, which plays a role in the present research context, since men have been found to describe themselves as less caring and more assertive [42, 43] and more alexithymic than women [10, 11]. Interestingly, in all previous studies concerning the association of alexithymia and interpersonal problems [29–36] the number of female participants clearly exceeded the number of male participants.

Recent comprehensive analyses of responses on the IIP confirmed that the latent structure of interpersonal problems is best represented by two continuous dimensions, which are largely independent of each other, agency and communion [44]. In our correlation analyses, we focus on these two interpersonal dimensions and the global index of severity concerning interpersonal problems (i.e., the total score of the IIP). Based on the results from previous patient-based studies [29, 31, 33] it was hypothesized that the alexithymia facets difficulties identifying feelings and difficulties in describing feelings are positively related to the global severity of interpersonal problems. Moreover, it was expected that difficulties identifying feelings, difficulties in describing feelings, and externally oriented thinking are positively related to the interpersonal problem domain cold (cf. [29, 35, 36]) and negatively related to the interpersonal dimension of communion [30]. Finally, it was assumed that difficulties identifying feelings and difficulties in describing feelings are positively related to the interpersonal problem domain nonassertive [29, 33] and against this backdrop we expected a negative correlation of these TAS-20 subscales with the interpersonal dimension of agency. We performed linear regression analyses to examine which alexithymia facets were associated independently from each other with interpersonal problem dimensions and global severity of interpersonal distress, after adjusting for negative affect.

## Methods

### Participants

The sample comprised 200 healthy adults (100 women and 100 men) with a mean age of 23.97 years (SD = 3.93, range: 18–35). Study participants were recruited through public notices posted in canteens, libraries, and public buildings of the University of Leipzig and through advertisements on several social media platforms (Facebook, Telegram, WhatsApp, “*Das schwarze Brett Leipzig*”, and the network “*Stud-IP*”). The majority of participants were university students enrolled in different faculties (79%). Their mean duration of school education was 12.15 years (SD = 0.71). All participants were native speakers of German. They gave consent to participate after full explanation of the investigation. At the start of the study, participants were interviewed via telephone by trained doctoral students about their mental health status and psychotherapeutic or psychiatric treatments and hospitalizations, which had a duration of about 30 min. During the self-developed screening interview, participants were asked whether they use psychotropic medication, alcohol, cannabis, or other substances and whether they suffer from depressed mood, anxieties, or symptoms of other mental disorders. Moreover, questions were asked about possible psychotherapeutic, psychiatric, or neurological (inpatient or outpatient) treatments and possible prescriptions of psychotropic medications by general practitioners. In case of any suspicion of a mental disorder, use of a psychotropic drug, or psychotherapeutic/psychiatric/neurological treatment the interview was terminated, and the participant excluded from the study. The study was approved by the ethics committee of the University of Leipzig, Medical School and performed in accordance with the Declaration of Helsinki. All participants were financially compensated for their involvement in the study. They received 20 € after completion of all tests. The payment of the participation fee was made by bank transfer.

### Measures

The *20-Item Toronto Alexithymia Scale* (TAS-20) is a self-report measure of alexithymic characteristics developed by Bagby et al. [5, 45] with proven validity and reliability (German version [46]. The TAS-20 consists of three subscales: a) Difficulties identifying feelings (DIF) with seven items, b) Difficulties describing feelings (DDF) with five items, and c) Externally oriented thinking (EOT) with eight items. The items are rated on a Likert scale ranging from 1 to 5, resulting in a maximum score of 100. Higher scores indicate a higher level of alexithymia. In addition to the subscale scores, a total score can be calculated by summing the twenty items.

The German version of the *Inventory of Interpersonal Problems* (IIP-D [47]) has eight subscales, with eight items on each subscale: Domineering (PA; i.e., difficulties in relaxing control over others), Vindictive (BC; i.e., difficulties of hostile dominance and the tendency to fight with others), Cold (DE; i.e., low degrees of affection for and connection with others), Socially Avoidant (FG; i.e., feelings of anxiety and avoidance in the presence of others), Nonassertive (HI; i.e., difficulties in taking initiative in relation to others and coping with social challenges), Exploitable (JK; i.e., excesses of friendly submissiveness), Overly Nurturant (LM; i.e., tendency to affiliate excessively), and Intrusive (NO; i.e., problems with friendly dominance). The inventory uses a 5-point scale ranging from 0 (not at all) to 4 (extremely) corresponding to different distressing interpersonal inhibitions or excesses. The IIP is a widely used clinical and research measure of interpersonal difficulties [25, 48, 49]. The IIP dimensionally assesses the nature of dysfunctional interpersonal patterns and provides a total score indicating the general degree of interpersonal problems. The psychometric properties of the German version of the IIP-D are similar to those of the original version [50].

In our study, we followed the scoring system for three interpersonal scores (a global index of interpersonal distress and the two interpersonal dimensions agency and communion) as described by Wendt et al. [44]:

$$\mathrm{General}\;\mathrm{Interpersonal}\;\mathrm{Distress}=\;(\mathrm{PA}\:+\:\mathrm{BC}\:+\:\mathrm{DE}\:+\:\mathrm{FG}\:+\:\mathrm{HI}\:+\:\mathrm{JK}\:+\:\mathrm{LM}\:+\:\mathrm{NO})\;/\;8.$$

$$Agency\:=\:PA\:+\:(NO\;x\;.71)\:+\:(BC\;x\;.71)-(FG\;x\;.71)-(JK\;x\;.71)-HI$$

$$Communion\:=\:LM\:+\:(NO\;x\;.71)\:+\:(JK\;x\;.71)-(BC\;x\;.71)-(FG\;x\;.71)-DE$$

The *Beck Depression Inventory* (BDI-II; German version [51]) is a short self-report questionnaire developed to measure the severity of depressive symptoms during the preceding two weeks. The BDI-II consists of 21 multiple-choice items, with 4 statements per item, that relate to symptoms such as, for example, pessimism, crying, irritability, and social withdrawal. Each item has four response options that range from 0 to 3.

The *State-Trait Anxiety Inventory* (STAI; German version [52]) is a brief self-report measure of current (state) and dispositional (trait) anxiety. The scales for state and trait anxiety consist of 20 items each to which subjects are asked to answer to what degree the items describe their situational or dispositional perceptions on a 4-point Likert-type scale (from 1 to 4).

The *Multiple-choice vocabulary intelligence* test (Mehrfachwahl-Wortschatz-Intelligenztest, MWT-B [53]) is a performance test, which measures aspects of general intelligence, specifically crystallized, verbal intelligence. The MWT-B comprises 37 items (i.e., rows) with increasing difficulty. Each item consists of four pronounceable pseudo-words and one real word. Subjects are asked to mark the real word without time restrictions. Raw scores (ranging from 0 to 37) can be converted to IQ scores.

### Procedure

This study was conducted at the Department of Psychosomatic Medicine and Psychotherapy, University of Leipzig. Due to the COVID-19 pandemic, all participants and the experimenter wore a face mask throughout the experiment. All subjects were tested individually in a quiet room. At the beginning of the experimental session, all study participants gave their informed written consent to participate in the study. The tests and questionnaires were administered in the following order: sociodemographic questionnaire, STAI-S, TAS-20, STAI-T, MWT-B, BDI-II, and IIP-D.

### Statistical analyses

Pearson correlation analysis was used to examine the relationships between TAS-20, IIP-D, measures of negative affectivity, and verbal intelligence. Correlation coefficients were calculated for the total sample as well as for men and women separately. Correlation coefficients between alexithymia and interpersonal problem dimensions were compared between men and women using *Z* statistics [54]. Hierarchical regression analyses were performed for each of the IIP-D dimensions/scores to examine the relationships of alexithymia scales with interpersonal problem dimensions and IIP-D total score, adjusting the effects of negative affectivity (i.e., current, and dispositional anxiety and current depression symptoms). The first step included STAI state, STAI trait, and BDI-II scores. In the second step, TAS-20 subscale scores were entered as predictors. To explore gender differences in alexithymia, interpersonal problems, anxiety, depression, and verbal intelligence *t*-tests for independent samples were administered. Statistical calculations were made with SPSS 27.0 (IBM Corp., Armonk, NY, USA), except the comparisons of correlation coefficients, which were computed using the software *Psychometrica* (https://www.psychometrica.de/korrelation.html [55]). Our main research question refers to the analysis of the relationships of the alexithymia facets with the two interpersonal problem dimensions and the global severity of interpersonal distress. We consider the correlation analysis between alexithymia facets and IIP-D subscales as secondary since it is based on the same set of questionnaire responses. However, the correlation analyses between TAS-20 scales and IIP-D scales help to better understand how the poles of the interpersonal dimensions contribute to significant correlations at the dimensional level. To adjust for multiple testing, we divided the standard level of significance *p* < 0.05 by the number of correlation calculations (*n* = 12, four TAS-20 scores x three IIP-D dimensional/total scores) necessary to answer the main research question, which led to a corrected significance threshold of *p* < 0.0042 (two-tailed). It should be noted that many statistical analyses (correlations and *t*-tests between women and men) were conducted to identify confounding variables that could be associated with alexithymia facets or interpersonal problems (i.e., state and trait anxiety, depression, verbal intelligence, and gender) and that should be controlled in our analyses. For these supplementary analyses, no p-level correction was applied. Here, results were considered significant at *p* < 0.05, two-tailed.

## Results

### Descriptive statistics

Mean scores, standard deviations, and ranges for the scales of the TAS-20, IIP-D, STAI, BDI-II, and MWT-B in the total sample are shown in Table 1. According to *t*-tests, women had higher DIF scores, *t*(198) = 2.24, *p* < 0.05, but lower EOT scores than men, *t*(198) = -2.72, *p* < 0.01 (see for details Table 2). Women did not differ from men concerning the DDF score and the total score of the TAS-20. For the IIP-D scales Nonassertive, Exploitable, and Overly nurturant higher scores were found in women compared to men, *t*(198) = 3.75, *p* < 0.001, *t*(198) = 4.17, *p* < 0.001, and *t*(198) = 2.68, *p* < 0.01 (see Table 2). Women had higher scores on the IIP-D scores General interpersonal distress and the dimension Communion, *t*(198) = 2.52, *p* < 0.05, and *t*(198) = 2.53, *p* < 0.05. In contrast, women had lower scores on the IIP-D dimension Agency compared to men, *t*(198) = -3.32, *p* = 0.001 (see Table 2). No significant gender differences were revealed for state anxiety (STAI), level of depressive symptoms (BDI-II), and verbal intelligence (MWT-B) (*p*s > 0.10) but women reported higher trait anxiety (STAI) than men, *t*(198) = 2.08, *p* < 0.05 (see Table 2).

**Table 1:** Descriptive statistics on psychological measures (*N* = 200, respectively)

	Mean	SD	Range
TAS-20 DIF	13.95	4.37	7–26
TAS-20 DDF	11.79	3.98	5–22
TAS-20 EOT	15.40	3.86	8–33
TAS-20 total score	41.14	9.11	21–71
IIP-D Domineering	6.16	4.14	0–19
IIP-D Vindictive	7.15	3.58	0–19
IIP-D Cold	8.49	5.18	0–21
IIP-D Socially Avoidant	9.96	5.59	0–25
IIP-D Nonassertive	12.95	5.80	1–28
IIP-D Exploitable	13.47	5.50	3–26
IIP-D Overly Nurturant	13.49	5.09	3–25
IIP-D Intrusive	10.16	4.89	0–25
IIP-D distress (IIP-D total score)	10.23	3.31	2.25–18.50
IIP-D agency (dimension)	-11.14	12.13	-43.46–24.07
IIP-D communion (dimension)	9.62	11.96	-23.91–44.69
STAI state	34.99	6.44	23–57
STAI trait	39.25	8.97	24–65
BDI-II	8.13	6.23	0–38
MWT-B IQ	110.44	11.13	93–145

*TAS-20* 20-Item Toronto Alexithymia Scale, *IIP-D* Inventory of Interpersonal Problems, *STAI* State Trait Anxiety Inventory, *BDI-II* Beck Depression Inventory, *MWT-B* Multiple-choice vocabulary test version B, intelligence quotient

**Table 2:** Descriptive statistics on psychological measures as a function of gender (*n* = 100, respectively)

	**Women**		**Men**	
	Mean	SD	Mean	SD
TAS-20 DIF	14.64	4.55	13.27	4.08
TAS-20 DDF	12.06	4.32	11.52	3.60
TAS-20 EOT	14.67	3.84	16.13	3.76
TAS-20 total score	41.37	9.37	40.92	8.88
IIP-D Domineering	5.78	3.76	6.54	4.46
IIP-D Vindictive	7.13	3.52	7.18	3.66
IIP-D Cold	8.67	5.27	8.31	5.10
IIP-D Socially Avoidant	10.25	5.83	9.68	5.34
IIP-D Nonassertive	14.44	5.75	11.46	5.48
IIP-D Exploitable	15.03	5.51	11.91	5.05
IIP-D Overly Nurturant	14.44	5.15	12.54	4.88
IIP-D Intrusive	10.75	4.57	9.57	5.15
IIP-D distress (IIP-D total score)	10.81	3.28	9.65	3.26
IIP-D agency (dimension)	-13.91	11.53	-8.36	12.13
IIP-D communion (dimension)	11.73	12.29	7.51	11.29
STAI state	34.80	6.40	35.19	6.51
STAI trait	40.56	8.67	37.94	9.11
BDI-II	8.51	5.99	7.75	6.46
MWT-B IQ	110.61	10.23	110.27	12.01

*TAS-20* 20-Item Toronto Alexithymia Scale, *IIP-D* Inventory of Interpersonal Problems, *STAI* State Trait Anxiety Inventory, *BDI-II* Beck Depression Inventory, *MWT-B* Multiple-choice vocabulary test version B, intelligence quotient

### Relationships of alexithymia with anxiety, depression, and verbal intelligence

From the alexithymia measures, the scales DIF and DDF as well as the total score of the TAS-20 were positively correlated with state and trait anxiety (STAI) and depressive symptoms (BDI-II) (see Table 3). In contrast, the EOT score was only correlated with state anxiety (STAI). None of the alexithymia measures was related to verbal intelligence (see Table 3).

**Table 3:** Pearson correlations of alexithymia scales (TAS-20) with state and trait anxiety, depression, and verbal intelligence

	**DIF**	**DDF**	**EOT**	**Total score**
STAI state	.28**	.26**	.22*	.34**
STAI trait	.53**	.42**	.13	.49**
BDI-II	.54**	.44**	.10	.49**
MWT-B IQ	.00	.00	.09	.04

*STAI* State Trait Anxiety Inventory, *BDI-II* Beck Depression Inventory, *MWT-B* Multiple-choice vocabulary test version B, intelligence quotient

*p < 0.01; ** *p* < 0.001 (two-tailed)

### Relationships of interpersonal problems with anxiety, depression, and verbal intelligence

All scales of the IIP-D showed positive correlations with the measures of negative affectivity (STAI and BDI-II), except the IIP-D scale Exploitable, which was not significantly correlated with STAI state (see Supplementary Table S1). Verbal intelligence was not related to any of the IIP-D scores. As could be expected, the IIP-D score General interpersonal distress was highly correlated with the measures of negative affectivity (STAI and BDI-II). The dimension Agency showed significant but lower (negative) correlations with the measures of negative affectivity (STAI and BDI-II). Finally, the dimension Communion did not correlate with any of the negative affect measures (see Supplementary Table S1).

### Relationships of alexithymia with interpersonal problems

All alexithymia measures were positively correlated with the IIP-D score General interpersonal distress (see Table 4). The scale DDF and the TAS-20 total score were negatively correlated with the IIP-D dimension Agency. Moreover, the scale DDF and the TAS-20 total score were negatively correlated with the IIP-D dimension Communion (see Table 4 for details). The scales DIF and DDF as well as the total score of the TAS-20 were positively correlated with almost all IIP-D subscales (see Table 4). The scale EOT was only correlated with the IIP-D subscales Vindictive, Cold, and Socially avoidant.

**Table 4:** Pearson correlations between alexithymia scales (TAS-20) and IIP-D scales and interpersonal dimensions (IIP-D) and partial correlations controlling for state anxiety (STAI), trait anxiety (STAI), and depressive symptoms (BDI-II) on the right of the slash

	**DIF**	**DDF**	**EOT**	**Total score**
IIP-D Domineering	.26**/.14	.16/.04	.07/.04	.23*/.10
IIP-D Vindictive	.35**/.19	.28**/.14	.25**/.22*	.39**/.26**
IIP-D Cold	.43**/.20*	.54**/.40**	.22*/.18	.54**/.37**
IIP-D Socially Avoidant	.48**/.21*	.56**/.41**	.28**/.25**	.59**/.41**
IIP-D Nonassertive	.43**/.20*	.33**/.13	.16/.10	.42**/.21*
IIP-D Exploitable	.26**/.10	.24**/.12	.05/.02	.25**/.11
IIP-D Overly Nurturant	.39**/.14	.26**/.04	.10/.05	.34**/.11
IIP-D Intrusive	.31**/.17	.00/-.19	.01/-.04	.15/-.03
**IIP-D distress (total score)**	.55**/.29**	.46**/.24**	.21*/.17	.56**/.33**
**IIP-D agency (dimension)**	-.19/-.04	-.31**/-.22*	-.10/-.07	-.27**/-.16
**IIP-D communion (dimension)**	-.08/-.04	-.29**/-.29**	-.18/-.17	-.24**/-.23**

*p < 0.0042 (corrected p-level); ** *p* < 0.001 (two-tailed, respectively)

We calculated additional correlations between TAS-20 and IIP-D scores for men and women, respectively (see Supplementary Table S2). For comparing the correlation coefficients between genders, we used *Z* statistics. In no case, significant differences were revealed between the correlation coefficients.

Three hierarchical regression analyses were conducted with the TAS-20 subscales as predictors and each of the IIP-D dimensions/scores as criterion variable controlling for negative affectivity. First, a regression model was calculated for interpersonal distress. In the first step, trait anxiety and current depressive symptoms were found to significantly predict interpersonal distress. In the second step, after the inclusion of the TAS-20 subscales, DIF was a significant positive predictor of interpersonal distress (see Table 5 for details). Second, we calculated a regression model for interpersonal agency. In the first step, trait anxiety was a significant negative predictor of agency. In step two, DDF was found to be a negative predictor of interpersonal agency (see Table 6). Finally, a regression model was calculated for interpersonal communion. In the first step, none of the negative affectivity measures predicted communion. In the second step, DDF was a negative predictor of interpersonal communion (see Table 7).

**Table 5:** Hierarchical regression predicting interpersonal *distress* (total score of the IIP-D) in two steps by state anxiety (STAI), trait anxiety (STAI), depression (BDI-II), and alexithymia scales (TAS-20) (*N* = 200)

	**Coefficients**				**Multicollinearity**		**Model**	
**Predictor**	**β**	**Beta**	***t***	**Sig. (*p*)**	**Tol**	**VIF**	**R**^**2**^	∆**R**^**2**^
**Step1**								
State anxiety	0.01	.02	0.29	.77	.72	1.39	.411*	-
Trait anxiety	0.18	.48	6.48	< .001	.55	1.82		
Depression	0.11	.21	3.08	.002	.63	1.59		
**Step 2**								
State anxiety	0.00	.00	0.07	.94	.70	1.43	.480*	.069*
Trait anxiety	0.14	.38	5.13	< .001	.50	2.01		
Depression	0.06	.10	1.50	.13	.55	1.82		
DIF	0.18	.23	3.31	.001	.54	1.83		
DDF	0.08	.09	1.40	.16	.60	1.66		
EOT	0.08	.09	1.71	.09	.87	1.14		

*β* unstandardized regression coefficient, *Tol.* Tolerance, *VIF* Variance Inflation Factor

*p < 0.001 (two-tailed)

**Table 6:** Hierarchical regression predicting the interpersonal dimension *agency* in two steps by state anxiety (STAI), trait anxiety (STAI), depression (BDI-II), and alexithymia scales (TAS-20) (*N* = 200)

	**Coefficients**				**Multicollinearity**		**Model**	
**Predictor**	**β**	**Beta**	***t***	**Sig. (*p*)**	**Tol**	**VIF**	**R**^**2**^	∆**R**^**2**^
**Step1**								
State anxiety	0.01	.01	0.10	.92	.72	1.39	.104**	-
Trait anxiety	-0.47	-.35	-3.85	< .001	.55	1.82		
Depression	0.09	.04	0.52	.60	.63	1.59		
**Step 2**								
State anxiety	0.03	.02	0.22	.83	.70	1.43	.149**	.045*
Trait anxiety	-0.42	-.31	-3.29	.001	.50	2.01		
Depression	0.19	.10	1.09	.27	.55	1.82		
DIF	0.16	.06	0.62	.53	.54	1.83		
DDF	-0.78	-.25	-2.98	.003	.60	1.66		
EOT	-0.02	-.01	-0.09	.92	.87	1.14		

*β* unstandardized regression coefficient, *Tol.* Tolerance, *VIF* Variance Inflation Factor

*p < 0.05; ** *p* < 0.001 (two-tailed)

**Table 7:** Hierarchical regression predicting the interpersonal dimension *communion* in two steps by state anxiety (STAI), trait anxiety (STAI), depression (BDI-II), and alexithymia scales (TAS-20) (*N* = 200)

	**Coefficients**				**Multicollinearity**		**Model**	
**Predictor**	**β**	**Beta**	***t***	**Sig. (*p*)**	**Tol**	**VIF**	**R**^**2**^	∆**R**^**2**^
**Step 1**								
State anxiety	-0.04	-.02	-0.26	.79	.72	1.39	.007	-
Trait anxiety	-0.05	-.04	-0.39	.70	.55	1.82		
Depression	-0.08	-.04	-0.47	.63	.63	1.59		
**Step 2**								
State anxiety	0.01	.01	0.10	.92	.70	1.43	.104*	.096**
Trait anxiety	0.01	.01	0.10	.92	.50	2.01		
Depression	0.03	.02	0.19	.85	.55	1.82		
DIF	0.29	.10	1.13	.26	.54	1.83		
DDF	-1.01	-.34	-3.83	< .001	.60	1.66		
EOT	-0.28	-.09	-1.26	.21	.87	1.14		

*β* unstandardized regression coefficient, *Tol.* Tolerance, *VIF* Variance Inflation Factor

*p < 0.01; ** *p* < 0.001 (two-tailed)

## Discussion

The present study examined the associations between alexithymia and interpersonal problems in healthy young adults. We conducted the study because there is scant research on this relationship. The only previous investigation based on a sample of healthy individuals [36] can be criticized since participants’ negative affects were not considered in the analyses. As mentioned above, negative affects may intensify relationship problems [39, 40] and seem to be associated with heightened levels of alexithymia [17, 41]. It seems worthwhile to examine healthy samples because the presence of a mental or somatic disease might affect type and severity of interpersonal problems as well as alexithymia and alter its relationship. Our correlational analyses were focused on the one hand on alexithymia facets as recent research findings underscore the importance of considering the alexithymia facets separately when investigating cognitive-emotional functioning [37, 38] and on the other hand on the two main dimensions of interpersonal problems (agency and communion) as assessed by the IIP and a global index of interpersonal problems [28, 44].

### Relationships of alexithymia with interpersonal problems

Our results indicate a large number of positive correlations between the TAS-20 and the IIP subscales, especially for the facets difficulties identifying feelings and difficulties verbalizing feelings and, to a lesser extent, for the facet externally oriented thinking. All TAS-20 subscales were positively associated with the global level of interpersonal problems. However, a more detailed examination revealed that, when controlling for negative affects, out of the TAS-20 subscales primarily difficulties identifying feelings predicted poor overall social adjustment and functioning (as assessed by the IIP). Thus, our findings suggest that in particular difficulties in identifying emotions and differentiating them from bodily sensations could be linked to overall levels of interpersonal distress. However, our zero-order and partial correlation results suggest that difficulties describing feelings might also play a role in poor social functioning. It must be noted in this context that in previous research difficulties identifying feelings emerged as a major predictor of a broad range of state psychopathology in psychiatric patients, whereas the contributions of the other facets were much smaller [56, 57]. It has been pointed out that individuals with difficulties in recognizing one’s emotions experience their emotional reactions in a rather undifferentiated manner [58]. They might, for example, be unsure if a negative feeling is fear, sadness, or shame. These individuals have subsequently less nuanced and accurate information available on which to base regulation strategies [59]. In this way, difficulties in identifying feelings might contribute to dysfunctions in affective regulation, which in turn could increase the risk for the development of psychopathological symptoms as well as interpersonal problems.

In our study, difficulties in identifying feelings, difficulties in describing feelings, and externally oriented thinking, as expected, were positively correlated with the interpersonal problem domain cold. Thus, all facets of alexithymia seem to be linked to interpersonal problems characterized by difficulties in feeling close to others and having affection for them. Only difficulties in describing feelings were found to be a negative predictor of the interpersonal problem dimension of communion independently from negative affect. This finding is basically in line with the results of Inslegers et al. [30] who did not analyze their data at the subscale level but reported a negative correlation between the global level of alexithymia and the IIP dimension communion in a mixed sample of patients and healthy individuals. This means that according to our data especially problems in the description, expression, and communication of emotions appear to be associated with tendencies to manifest excessive interpersonal distance characterized by being uncooperative, socially avoidant, and uncaring. It seems that the alexithymic facet difficulties in verbalizing and expressing emotions could represent a factor causing or exacerbating interpersonal problems or conflicts associated with social distance. It has been shown that the sharing of emotional experiences can be important for building and enhancing interpersonal closeness [60]. The specific interpersonal dynamic, which develops in the social sharing of emotion lends itself well to the strengthening of relational bonds [61]. A person sharing an emotional experience in a conversation arouses interest and emotions in the recipient (or listener). Their reciprocal stimulation of emotion can set both partners in a similar emotional state. As the recipient shows interest, and empathy, the disclosing person experiences enhanced liking for the recipient [62]. Thus, communicating one’s emotions to another person can lead to experiences of emotional communion, which is likely to strengthen social ties and to elicit support [61].

Confirming our assumption, difficulties identifying feelings and difficulties describing feelings were found to be positively related to the interpersonal problem domain nonassertive. However, at the dimensional level our data indicate that, when adjusting for negative affects, only difficulties describing feelings predicted low agency. The latter result partly confirms our hypothesis. This means, that difficulties in verbalizing and expressing one’s emotions appear to be linked to low dominance, i.e., submitting to others, being introverted and problems with being non-assertive. Possibly, deficits in describing and communicating one’s emotions may have an adverse effect on the negotiation of social hierarchies. In this context, it is not surprising that alexithymia has been found to be linked to low socioeconomic status [10, 13]. Deficits in describing and communicating emotions may interfere with the development of socially competent self-assertion strategies and may pose risks for exploitation and dependence in social relationships. Emotions inform oneself and others about the frustration or achievement of goals, behavioral intentions, coping resources, and attitudes [63, 64]. Thus, expressions of emotions do not only communicate characteristics about internal states or intentions of a sender, but they also are a means of directing other people's behavior [65]. In this sense, verbally or nonverbally expressed emotions can help to get a recipient to do something for the sender fulfilling his or her requests and expectations. People with deficits in emotion communication should be less assertive in interpersonal interactions and have less social success and impact.

The present findings are partly in line with those of the only previous investigation [36] which was based on a sample of healthy individuals. The correlations of the subscale Difficulties describing feelings observed in our study (when adjusting for negative affects) correspond in part to those found by Weinryb et al. [36]: difficulties in describing feelings were positively linked to the domains cold and socially avoidant. It appears that the relationship of difficulties describing feelings with a specific domain of interpersonal problems, i.e., low communion, could be quite robust in healthy individuals. Our findings concerning the alexithymia facet difficulties identifying feelings are in contrast with those of Weinryb et al. [36]: in our study there were many positive correlations between difficulties identifying feelings and interpersonal problems as assessed by the IIP whereas in that of Weinryb et al. [36] no correlations between these variables were revealed. Since the authors did not report the descriptive statistics of the TAS-20 scales in their study, it remains unclear whether the non-correlations of the facet difficulties identifying feelings could be due to a low mean and/or a low variance of the score.

### Effect of gender on the relationship between alexithymia and interpersonal problems

In our study, we explored the effect of gender on the relationship between facets of alexithymia and interpersonal problems. Gender could play an important role in the present research context, because men score higher, on average, than women on measures of alexithymia in clinical and nonclinical samples (see [66] for a meta-analysis). In addition, there is evidence that women describe themselves as more caring and less assertive compared to men [42, 43]. Thus, there appear to be differences in the extent of interpersonal problems between genders. Even though in our sample we observed higher communion and lower agency in women compared to men corroborating previous findings, the correlations between alexithymia facets and interpersonal problems did not differ between genders. Thus, it seems that at least in healthy samples the relationships between alexithymia and interpersonal problems may not vary as a function of gender.

### Limitations of the study

Several limitations should be considered when interpreting our results. We are aware of the criticism that has been directed at self-report scales of alexithymia like the TAS-20. It has been argued that alexithymic individuals may not be able to make valid evaluations about their own deficits in perceiving and dealing with emotions [67]. Against this background, it has been proposed [6, 68, 69] that in alexithymia research self-report instruments should be combined with interview-based or observer-rated measures such as the Toronto Structured Interview for Alexithymia (TSIA [70]). However, it should be noted that in several previous studies on alexithymia and emotion processing in which self-report tests were combined with interviews or observer-ratings of alexithymia self-report data were observed to be better predictors of neurocognitive processes of emotion perception than the scores derived from interview or observer rating [71–73]. It can be assumed that alexithymic individuals receive negative feedback from the social environment concerning their deficits in recognizing, expressing, and communicating their emotions. In this way, alexithymic individuals can become aware of their own deficits and integrate this information into their self-concept.

We must be careful with generalizing our results since our sample consisted of young, well-educated, healthy individuals. It is necessary to replicate our results in populations other than college students before strong conclusions can be made. Given that there are cultural differences in terms of how people of different cultures behave socially and experience emotions, it is advisable to conduct studies on the subject in other cultural areas. Furthermore, it is recommended to administer standardized clinical interviews such as the SCID-5-CV [74] in future studies to exclude presence of mental disorders in study participants instead of self-developed screening interviews, which we administered in our investigation. Standardized clinical interviews allow researchers also to record and assess much more details concerning participants’ substance use and abuse. In the present study, data on participants’ mental health status and medication came exclusively from self-report. Future research should use medical records to verify the information provided by participants. In our study, individuals received a fee for their participation. It can be criticized that monetary compensation may introduce biases in the recruitment process and reduce the representativeness of a sample. Therefore, financially needy individuals from disadvantaged social backgrounds can be overrepresented in our sample. Finally, the overall number of participants in our study was rather low, especially for a comparison between genders. To detect small differences between genders in the relationships between alexithymia and interpersonal problems it seems necessary to recruit larger subsamples.

## Conclusions

Our study investigating the links between alexithymia and interpersonal problems in healthy subjects provides evidence that specific alexithymia facets could affect specific processes of interpersonal functioning. We found primarily difficulties in identifying emotions (but also difficulties in describing feelings) to be associated with high overall levels of interpersonal distress. It may be hypothesized that difficulties identifying feelings disrupt emotion regulation processes that in turn could heighten the general risk of interpersonal and other mental problems. A positive association between difficulties in identifying emotions and overall levels of interpersonal distress has also been reported in studies based on patient samples [29, 31, 33]. Moreover, the facet difficulties describing feelings was found to be an important negative predictor of the interpersonal problem dimensions communion and agency independently from negative affects. Problems in the verbalization and communication of one’s emotions could be of particular importance in the development and maintenance of interpersonal problems related to low communion and low agency. As pointed out above, the expression of emotions is a key factor in establishing experiences of social closeness as well as in directing other people’s behavior. The latter function helps to assert one's interests. Impairments in emotion expression should contribute to social isolation as well as non-assertiveness. Interestingly, this finding is in line with a prediction from a recent integrative review on emotional-cognitive processing in alexithymia [37] that especially the alexithymia facet difficulties describing feelings, reflecting deficits in expressing and communicating emotion to others, should have an adverse impact on interpersonal relationships. Based on the results of our and previously published studies [29, 31, 33, 35, 36], it can be concluded that the alexithymia facet externally oriented thinking is only weakly, if at all, associated with interpersonal problems. Because correlation does not indicate causation longitudinal research is required to establish whether difficulties describing feelings may be causative for interpersonal problems linked to social isolation and non-assertiveness. To this aim, longitudinal training interventions could be conducted focused on the elaboration and expression of emotions. It would be important to assess whether such trainings could reduce not only alexithymic features but also interpersonal problems.

In view of our differential correlation findings, the use of a dimensional approach with the calculation of IIP scores for communion and agency appears to be promising in future clinical studies on alexithymia and interpersonal problems. As all of the previous clinical investigations on the subject examined samples of patients suffering from various psychiatric or somatic symptoms and diseases [29, 31–33, 35] future research may include homogenous clinical samples to investigate the impact of specific disorders on the relationship between alexithymia and interpersonal problems.

### Supplementary Information

**Additional file 1: Supplementary Table S1. **Correlations of anxiety, depression, and verbal intelligence with IIP-D scales and interpersonal dimensions (IIP-D) (*N* = 200). **Supplementary Table S2.** Correlations between alexithymia scales (TAS-20) and IIP-D scales and interpersonal dimensions (IIP-D) for women (coefficient before the slash) and men (coefficient after the slash) (*n* = 100, respectively).

## Data Availability

The datasets used and/or analyzed during the current study are available from the corresponding author on reasonable request.

## Abbreviations

BC: Subscale Vindictive of the IIP-D
BDI-II: Beck Depression Inventory
DE: Subscale Cold of the IIP-D
IIP-D: Inventory of Interpersonal Problems, German version
DIF: Difficulties Identifying Feelings
DDF: Difficulties Describing Feelings
EOT: Externally Oriented Thinking
FG: Subscale Socially Avoidant of the IIP-D
HI: Subscale Nonassertive of the IIP-D
JK: Subscale Exploitable of the IIP-D
LM: Subscale Overly Nurturant of the IIP-D
MWT-B: Multiple-choice vocabulary test version B
NO: Subscale Intrusive of the IIP-D
PA: Subscale Domineering of the IIP-D
STAI: State-Trait Anxiety Inventory
TAS-20: 20-Item Toronto Alexithymia Scale
TSIA: Toronto Structured Interview for Alexithymia
