# Supplementary material for: Alexithymia and interpersonal problems in healthy young individuals
Source: BMC Psychiatry. 2023 Sep 21;23:688. doi: 10.1186/s12888-023-05191-z (PMC10515237; doi:10.1186/s12888-023-05191-z)
Supplement: Supplementary file 1 — Additional file 1: Supplementary Table S1. Correlations of anxiety, depression, and verbal intelligence with IIP-D scales and interpersonal dimensions (IIP-D) (N = 200). Supplementary Table S2. Correlations between alexithymia scales (TAS-20) and IIP-D scales and interpersonal dimensions (IIP-D) for women (coefficient before the slash) and men (coefficient after the slash) (n = 100, respectively). [file 12888_2023_5191_MOESM1_ESM.docx]

Supplementary Table S1

Correlations of anxiety, depression, and verbal intelligence with IIP-D scales and interpersonal dimensions (IIP-D) (N = 200).

STAI state STAI trait BDI-II MWT-B IQ

-----------------------------------------------------------------------------------------------------------------

IIP-D Domineering .14* .22** .25*** -.11

IIP-D Vindictive .18* .38*** .25*** -.02

IIP-D Cold .27*** .42*** .45*** -.07

IIP-D Socially Avoidant .34*** .59*** .45*** .05

IIP-D Nonassertive .31*** .53*** .34*** .03

IIP-D Exploitable .13 .32*** .24*** .04

IIP-D Overly Nurturant .24*** .47*** .39*** -.06

IIP-D Intrusive .21** .27*** .29*** -.06

IIP-D distress (total score) .35*** .62*** .51*** -.03

IIP-D agency (dimension) -.15* -.32*** -.16* -.11

IIP-D communion (dimension) -.06 -.07 -.07 -.01

-----------------------------------------------------------------------------------------------------------------

* *p* < 0.05; ** *p* < 0.01; *** *p* < 0.001 (two-tailed). IIP-D: Inventory of Interpersonal Problems; STAI: State Trait Anxiety Inventory; BDI-II: Beck Depression Inventory; MWT-B: Multiple-choice vocabulary test version B, intelligence quotient.

Supplementary Table S2

Correlations between alexithymia scales (TAS-20) and IIP-D scales and interpersonal dimensions (IIP-D) for women (coefficient before the slash) and men (coefficient after the slash) (n = 100, respectively)

DIF DDF EOT Total score

-----------------------------------------------------------------------------------------------------------------

IIP-D Domineering .33/.24 .14/.21 .14/-.01 .28/.19

IIP-D Vindictive .26/.46 .23/.34 .28/.22 .34/.44

IIP-D Cold .40/.48 .52/.57 .25/.21 .54/.54

IIP-D Socially Avoidant .51/.43 .63/.47 .20/.40 .62/.56

IIP-D Nonassertive .38/.44 .35/.29 .22/.23 .43/.42

IIP-D Exploitable .27/.19 .26/.20 .16/.06 .31/.19

IIP-D Overly Nurturant .42/.31 .28/.21 .12/.16 .38/.30

IIP-D Intrusive .37/.23 .00/-.02 .11/-.03 .22/.08

IIP-D distress (total score) .56/.52 .48/.43 .27/.24 .61/.51

IIP-D agency (dimension) -.20/-.13 -.40/-.20 -.10/-.21 -.32/-.23

IIP-D communion (dimension) -.04/-.20 -.28/-.35 -.10/-.20 -.19/-.32

-----------------------------------------------------------------------------------------------------------------
